# Mandibular talon cusps: A Systematic review and data analysis

**DOI:** 10.4317/jced.51476

**Published:** 2014-10-01

**Authors:** Sreekanth-Kumar Mallineni, George-Kurian Panampally, Yong Chen, Tian Tian

**Affiliations:** 1 Pedodontics and Preventive Dentistry, SDDCH, Parbhoni, Maharashtra, India; 2 Orthodontic department, Xiamen Dental Hospital, Xiamen City, China; 3 PhD candidate, University of Hong Kong, Hong Kong

## Abstract

Objectives: The purpose of the present study was to evaluate mandibular talon cusps distribution from the comprehensive literature search and proposal of new classification Material and Methods: The study was a review of articles published in the English language from January 1960 to December 2013. The PubMed/MEDLINE/Google Scholor databases were searched electronically using ‘talon cusp’, ‘dens evaginatus’, ‘anterior teeth’, mandible, ‘primary dentition’ and ‘ permanent dentition’ as search terms in various combinations. The citation lists from the included references were subsequently examined, and a hand search was also performed in an attempt to identify additional reports. The distribution, characteristics, common tooth type, associated dental anomaly and proposal of new classification have been included in final data analysis. Descriptive statistics were carried out using Chi square test (SPSS, version 17). Results: Overall 37 citations were retrieved from the literature where one was prevalence studies and rest were case reports among those two were duplication. Total 35 articles with 43 patients were reported on mandibular talon cusps. Males were predominantly affected than females (*p*<0.05). Eight cases (19%) were reported in archeological skulls 81% were clinical reports. Forty cases (93%) were reported in permanent dentition while 7% cases in primary dentition. Lingual mandibular talon are more common than facial type in permanent dentition facial talons (*p*<0.05). Seven cases (18%) were bilaterally involved. Double tooth (45%) was commonly associated with mandibular talons. Most of the cases reported from Asia and asia derived populations. Conclusions: Central incisor is the most common tooth type that effected by talon cusp in permanent dentition and lateral incisor is in primary dentition. Lingual talons are common in mandible. Double tooth common dental anomaly associated with mandibular talon cusp. Most of the case reported from Asia. Talon cusps should be classified as facial, lingual, and facial and lingual types.

** Key words:**Double tooth, permanent dentition, primary dentition, mandibular arch, Talon cusp.

## Introduction

Talon cusp is a cusp-like projection or tooth like from the palatal or labial surface of an anterior tooth that extended at least half the distance from the cement-enamel junction to the incisal edge contains enamel, dentin and/or pulp in maxillary or mandibular arch. Talon cusps are a rarely reported developmental abnormality of uncertain etiology that affects the anterior teeth in both dentitions. A curved horn-like process (1) and supernumerary cusps (2) on the palatal surfaces of permanent maxillary central incisors were first reported in the literature almost 120 years ago. Subsequently, various names like accessory cusp, cusped cingulum, dens evaginatus, evaginated odontome, horn, hyperplastic cingulum, supernumerary cusp and supernumerary lingual tubercle have been given by different authors for this portent (3). It has also been referred to as a cusp-like projection, hyperplasia of the cingulum, palatal accessory cusp and unusual projection of the facial surface of the anterior teeth (4). Due to its resemblance to the shape of an eagle’s talon Mellor and Ripa (5) called this anomaly as talon cusp. Earlier, there have been confusion among talon cusp and dens evaginatus. However, both are projections covered by enamel that contains pulp tissue and it is possible that a talon cusp could be the ultimate expression of a dens evaginatus, hence, the term “dens evaginatus of the anterior teeth” has also been used for “talon cusp” (3,4).

The majority of the studies on the prevalence of talon cusp have been reported in the permanent dentition and rarely on the primary dentition (3,6). al-Omari and colleagues (7) also revealed that permanent teeth are affected with talon cusp three times more frequently than primary teeth, and males are more commonly affected than females (3). The etiology of a talon cusp is also unknown; genetic and/or environmental factors may cause the cusp to develop (3,4,8). Similar to other defects in tooth form, a talon cusp originates during the morphodifferentiation stage of tooth development (9). Although, some of the reported variations may only reflect the use of different diagnostic criteria, or non-representative samples, data from carefully selected ethnically representative populations can provide useful data for at least some of these dental characteristics. The reported prevalence is between 0.06% and 7.7% (3,8). It is more common in the permanent dentition than in the primary dentition. Maxillary teeth more commonly effected in both dentitions, and maxillary lateral incisor is commonly affected in permanent dentition while central incisor in primary dentition (3,6,8,9). The occurrence of talon cusps in mandibular arch is very rare. Furthermore, there is no data available on characteristics of mandibular talon cusps. Therefore, the purpose of the present study was to evaluate mandibular talon cusps distribution from the com-prehensive literature search and proposal of new classification.

## Material and Methods

The study was a review of articles published in the English language from January 1960 to December 2013. The PubMed/MEDLINE database was searched electronically using ‘Talon cusp’, ‘dens evaginatus’, ‘anterior teeth’, mandible, ‘primary dentition’ and ‘ permanent dentition’ as search terms in various combinations. The citation lists from the included references were subsequently examined, and a hand search was also performed in an attempt to identify additional reports. Hand searching of all identified articles was used to supplement the electronic search. The reference lists of these articles were further checked to identify any other articles relevant to the mandibular talon cusps. Inclusion criteria include mandibular talon cusps, primary and permanent dentition, human subjects, anterior teeth, densevaginatus of anterior teeth and search was limited to English language only. The reports published other than English, talon cusps of posterior teeth, maxillary arch, duplications and case reports and studies on animal subjects were excluded. Data retrieved from the articles was divided into gender distribution, type of tooth, talon cusp associated with dental anomalies, and region of the mandible for evaluation of the reports that have been published. The distribution, characteristics, common tooth type, associated dental anomaly and proposal of new classification have been included in final data analysis. Descriptive statistics carried among males and female, and lingual and labial mandibular talons using chi square test [SPSS,version17].

## Results

**Figure 1:**
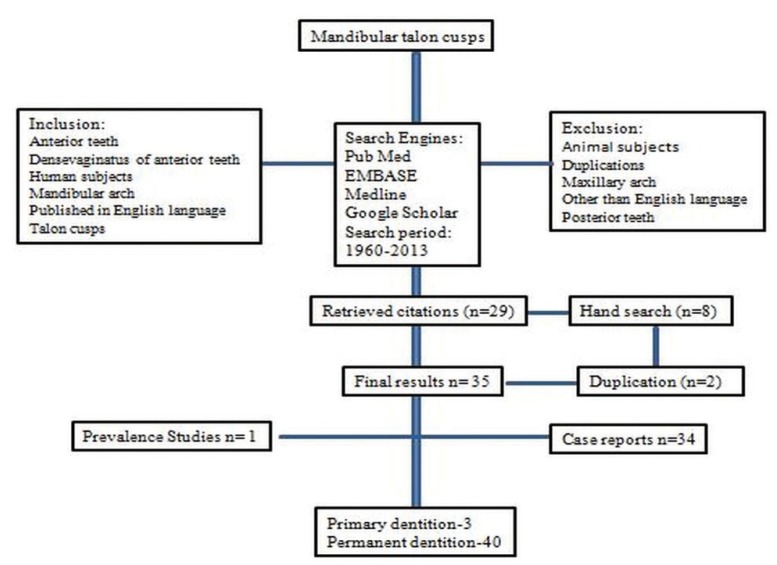
Study design flow chart.

The searches yielded a total of 256 citations and review of the titles and abstracts resulted in the exclusion of 204 of these studies and clinical reports. On further review of the remaining 53 complete papers, 16 were excluded after they were read in full. Overall 37 citations were retrieved from the literature that published on mandibular talon cusps among these two were duplications, which was mention in report separately (Fig. 1). Total 35 articles with 43 patients were reported on mandibular talon cusp in the published literature among those one was prevalence study and rest were case reports. At the present time, a total of 43 individuals have been observed with at least one mandibular talon cusp ( Table 1). Mandibulars talon cusps are more common in males than females (*p*<0.05) with 7:3 ratio. Eight cases [19%] were reported in archeological skulls where rest of the cases [81%] were clinical reports. Forty cases [93%] were reported in permanent dentition while three cases [7%] in primary dentition ( Table 1).

**Table 1:**
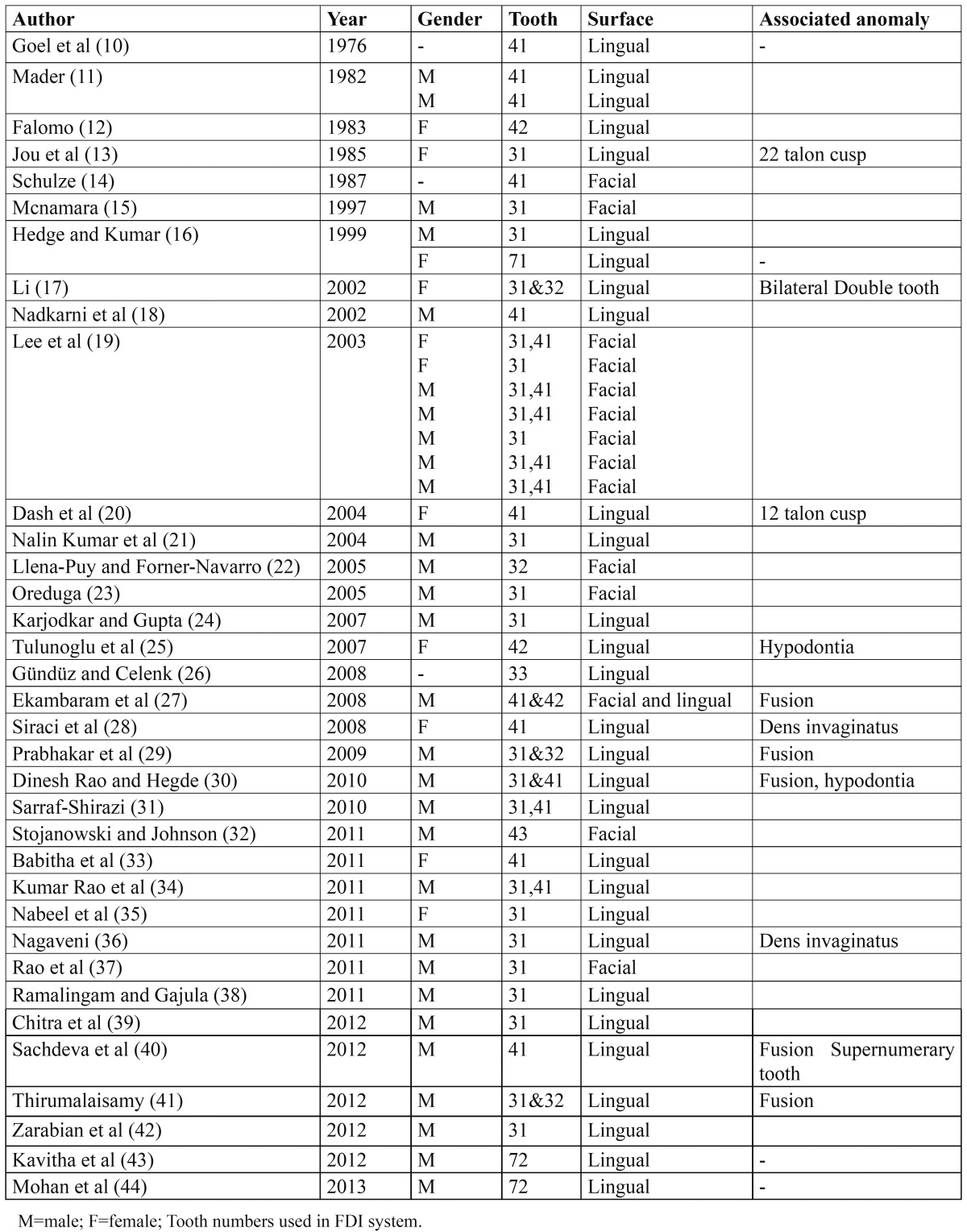
Documented cases of mandibular talon cusps reported in primary and permanent dentition.

**Figure 2:**
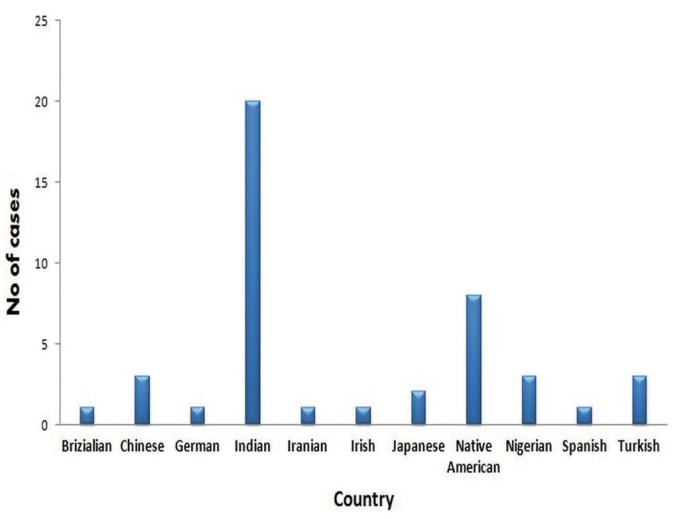
Distribution of mandibular talon cusps based on ethnicity.

Lingual talons are more common in mandible than facial talons in permanent dentition, where 12 cases [30%] were involved on facial surface, twenty-seven cases [68%] were on lingual surface, and one case [2%] exhibited on both surfaces. Only seven cases [17.5%] were bilaterally involved, rest were unilateral, among those thirty six were central incisors, three were lateral incisors and two were canines. In primary dentition only three cases involved among those one was central incisor [34%] and two lateral incisor [66%]. Comparatively left side incisors were involved more commonly than right side. Eleven cases [29%] in permanent dentition were associated with dental anomalies among those double tooth [45%] was common anomaly that associated with mandibular talon cusp. Most of the cases on mandibular talon cusps were reported from Asia, among those 90% case are from India (Fig. 2).

## Discussion

Among reported mandibular talon cusps 81% of the cases [81%] were reported in modern population and 19% were reported based on archeological available skulls. Surprisingly, all those cases that have been reported in skulls were facial talon cusps. It has been suggested that 75% of the talon cusp cases are in the permanent dentition, while, 25% in the primary dentition (8,9). Similarly, majority of the reported mandibular talon cusps involved permanent dentition [93%], while, three cases in primary dentition [7%]. Talon cusps in permanent teeth are commonly affected than primary teeth and even in mandibular arch similar to maxillary arch. In permanent dentition, seven cases [17.5%] were effected bilaterally, while, 82.5% cases were unilateral in presentation. Unilateral talon cusps are more common than bilateral occurrence in the mandibular arch similar to the maxillary arch. The three cases that reported in the primary dentition were unilateral in presentation. Among, those 13 [33%] cases of talon cusp involved the facial surface on the mandibular incisors alone and 26 [65%] cases of talon cusp involved the lingual surface of incisors in the manidibular arch. One known case [2%] exhibited a facial and a lingual talon cusp on the same tooth in mandibular arch (27). In maxillary teeth, talon cusp more commonly effected in both dentitions, and maxillary lateral incisor is commonly affected in permanent dentition while central incisor in primary dentition (7-9). Contrarily, our comprehensive review on mandibular talon cusps revealed central incisor is the common tooth type that exhibited talon cusp in permanent dentition, while lateral incisor in primary dentition.

The present survey on mandibular talon cusps evaluated that one third of the total cases associated with associated at least one dental anomaly. Among, those double tooth was the more frequently reported anomaly [45%], where one instance [9%] was associated with central incisor fused with supernumerary tooth and in two cases mandibular talon cusps were associated with maxillary talon cusp [18%]. It is very interesting that our results showed double tooth is the common dental anomaly that associated with mandibular talon cusp. Although, some of the reported variations may only reflect the use of different diagnostic criteria, or non-representative samples, data from carefully selected ethnically representative populations can provide useful data for at least some of these dental characteristics. The distribution of the mandibular talon cusps were highest among Asians. It was evident that majority of mandibular talon cusps clinical and archaeologically comes from Asian and Asia-derived populations, while the present review of cases illustrated that the global distribution of talon cusps among populations, both modern and archeological from Africa, Europe and North America.

In permanent dentition 12 cases [30%] were involved on facial surface, twenty-seven cases [68%] were on lingual surface, and one case [2%] reported on both surfaces. Based on this talon cusps should classify as facial, lingual, facial and lingual, and in present study it was evident that lingual talon cusps [65%] are common in mandible. Prior to this four classifications were proposed, based extension of cusp (8), shape (9), cingulam shape (45), and anomaly of the involved tooth (35).

The complications of talon cusp include aesthetic, diagnostic, functional, and pathological (5,11) variations. A large talon cusp is unaesthetic and presents clinical problems and it may present diagnostic problems if it is unerupted and resembles a compound odontome or a supernumerary tooth and so leads to unnecessary surgical procedure. Functional complications include displacement of teeth, occlusal interference, speech problems and trauma to the lip and tongue. The deep grooves which join the cusp to the tooth may also act as stagnation areas for plaque and debris, become carious and cause subsequent periapical pathology (5).

Management of talon cusp will depend on its presentation and complications. Generally, trace talons are asymptomatic and left without treatment (8), if, deep grooves are present prophylactic measures like fissure sealant and composite resin restoration should be planned (15,23). Especially, in case of occlusal interference, reduction of the bulk of the cusp gradually and periodically and application of topical fluoride application to reduce sensitivity and stimulate reparative dentine formation for pulp protection (46), or complete reduction of the cusp and calcium hydroxide pulpotomy (47). Root canal therapy is also an option sometimes extirpate where entire reduction of cusp and pulp extirpation is necessary (48). Orthodontic correction may become necessary when there is tooth displacement or malalignment or infra-occlusion of opposing teeth (15).

The mandibular talon cusp is rare and found in less than one percent of the population. This study presents forty three cases of the rare mandibular talon cusp and the first estimates of population frequencies. Mandibular talon cusps have been found on all mandibular anterior teeth [Incisors and cuspids]. To date, very little evidence indicates a direct relationship between the mandibular talon cusp described here and the more common maxillary talon cusp. Optimistically, future research will enable the researchers to better understand the etiology and genetic basis of this trait, as well as any possible correlation that may relate to other morphological features of the human dentition.

## Conclusions

As a number of cases of mandibular talon cusps have now been published, this study presents a survey of cases in the literature to characterize the expression of this rare trait, and compares it with that of maxillary talon cusps. Mandibular talon cusps are commonly seen in males than females. Permanent dentition is commonly affected than the primary dentition. Central incisor is the most common tooth type in mandibular arch that effected by talon cusp in permanent dentition and lateral incisor is in primary dentition. Talon cusps should be classified as facial, lingual, and facial and lingual types. One third of the mandibular talon cusp may be associated with dental anomalies where double tooth frequently reported. Most of the mandibular talons were reported from Asia and Asia derived populations among those 90% of the cases from India.

## References

[1] Mitchell WH (1892). Letter to editor. Dent Cosmos.

[2] Windle BCA, Humphreys J (1887). Extra cusps on the human teeth. Anat Anz.

[3] Lee CK, King NM, Lo EC, Cho SY (2006). Talon cusp in the primary dentition:literature review and report of three rare cases. J Clin Pediatr Dent.

[4] Mallineni SK, Manan NM, Lee CK, King NM (2013). Talon cusp affecting primary dentition in two siblings:a case report. Rom J Morphol Embryol.

[5] Mellor JK, Ripa LW (1970). Talon cusp:a clinically significant anomaly. Oral Surg Oral Med Oral Pathol.

[6] Hattab FN, Yasin OM, Al-Nimri KS (1996). Talon cusp in the permanent dentition associated with other dental anomalies:Review of literature and report of seven cases. J Dent Child.

[7] al-Omari MA, Hattab FN, Darwazeh AM, Dummer PM (1999). Clinical problems associated with unusual cases of talon cusp. Int Endod J.

[8] Dankner E, Harari D, Rotstein I (1996). Dens evaginatus of anterior teeth. Literature review and radiographic survey of 15,000 teeth. Oral Surg Oral Med Oral Pathol Oral Radiol Endod.

[9] Davis PJ, Brook AJ (1985). The presentation of talon cusp:diagnosis, clinical features, associations and possible aetiology. Br Dent J.

[10] Goel VP, Rohtagi VK, Kaushik KK (1976). Talon cusp:a clinical study. J Indian Dent Ass.

[11] Mader CL (1982). Mandibular talon cusp. J Am Dent Ass.

[12] Falomo OO (1983). Talon cusp:a case report. Odonto-stomatol Trop.

[13] Jou YT, Chuang TH, Lan WH, Kwan HW (1985). Talon cusp-report of cases. Zhonghua Ya Yi Xue Hui Za Zhi.

[14] Schulze C (1991). Anomalien und Mißbildungen der menschlichen Zähne. Berlin:Quintessenz Verlags- GmbH, 1987. Cited by Tsutsumi T, Oguchi H. Labial talon cusp in a child with in-continentia pigmenti achromians:case report. Pediatr Dent.

[15] McNamara T, Haeussler AM, Keane J (1997). Facial talon cusps. Int J Paediatr Dent.

[16] Hegde S, Kumar BR (1999). Mandibular talon cusps:report of two cases. Int J Paediatr Dent.

[17] Li RW (2002). Clinical variants in tooth number and crown form:a report of bilateral double teeth associated with a talon cusp. Dent Update.

[18] Nadkarni UM, Munshi A, Damle SG (2002). Unusual presentation of talon cusp:Two case reports. Int J Paediatr Dent.

[19] Lee C, Burnett SE, Turner CG (2003). Examination of the rare labial talon cusp on human anterior teeth. Dent Anthropol.

[20] Dash JK, Sahoo PK, Das SN (2004). Talon cusp associated with other dental anomalies:a case report. Int J Paediatr Dent.

[21] Nalin Kumar S, Ranganathan K, Umadevi M, Joshua E, Saraswathi TR (2004). Talon cusp:an overview with case reports of 3 clinical variants. Indian J Dent Res.

[22] Llena-Puy MC, Forner-Navarro L (2005). An unusual morphological anomaly in an incisor crown. Anterior dens evaginatus. Med Oral Patol Oral Cir Bucal.

[23] Oredugba FA (2005). Mandibular facial talon cusp:case report. BMC Oral Health.

[24] Karjodkar FR, Gupta A (2007). Mandibular talon cusp:a case report. Oral Surg Oral Med Oral Pathol Oral Radiol Endod.

[25] Tulunoglu O, Cankala DU, Ozdemir RC (2007). Talon's cusp:report of four unusual cases. J Indian Soc Pedod Prev Dent.

[26] Gündüz K, Celenk P (2008). Survey of talon cusps in the permanent dentition of a Turkish population. J Contemp Dent Pract.

[27] Ekambaram M, Yiu CKY, King NM (2008). An unusual case of double teeth with facial and lingual talon cusps. Oral Surg Oral Med Oral Pathol Oral Radiol Endod.

[28] Siraci E, Gungor HC, Cehreli ZC (2008). Dens invaginatus and talon cusp co-occurring in a mandibular central incisor:a case report. J Dent Child (Chic).

[29] Prabhakar AR, Kaur T, Nadig B (2009). Bilateral fusion of permanent mandibular incisors with Talon's cusp:A rare case report. J Oral Maxillofac Pathol.

[30] Dinesh Rao B, Hegde S (2010). A talon cusp on fused teeth associated with hypodontia:report of a unique case. Eur J Dent.

[31] Sarraf-Shirazi A, Rezaiefar M, Forghani M (2010). A rare case of multiple talon cusps in three siblings. Braz Dent J.

[32] Stojanowski CM, Johnson KM (2011). Labial canine talon cusp from the Early Holocene site of Gobero, central Sahara Desert, Niger. Int J Osteoarchaeol.

[33] Babitha GA, Shobha P, Mithul M (2011). Mandibular Talon Cusp. Int J Dent Assoc.

[34] Kumar Rao P, Ram Shetty SV, Prabhu R, Veena KM, Chatra L, Shenai P (2011). Talon cusps in mandibular incisors: an unusual presentation in a child patient. J Dent Res Dent Clin Dent Prospects.

[35] Nabeel S, Hegde U, Mull P, Danish G (2011). Talon cusp affecting two generations:report of two cases and proposed comprehensive classification. Int J Oral Maxillofac Pathol.

[36] Nagaveni NB, Umashanikara KV, Vidyullatha BG, Sreedevi S, Radhika NB (2011). Permanent mandibular incisor with multiple anomalies - report of a rare clinical case. Braz Dent J.

[37] Rao PK, Mascarenhas R, Shetty SR (2011). Facial talon in mandibular incisor:An unusual occurrence. Dent Res J (Isfahan).

[38] Ramalingam K, Gajula P (2011). Mandibular talon cusp:A rare presentation with the literature review. J Nat Sci Biol Med.

[39] Chaitra TR, Goswami M, Chaudhary S, Kulkarni A (2012). Mandibular talon's cusp. BMJ Case Rep.

[40] Sachdeva GS, Malhotra D, Sachdeva LT, Sharma N, Negi A (2012). Endodontic management of mandibular central incisor fused to a supernumerary tooth associated with a talon cusp:a case report. Int Endod J.

[41] Thirumalaisamy E, Baskaran P, Jeyanthi K, Kumar S (2012). Talon cusp in fused teeth:A rare concomitant occurrence. J Oral Maxillofac Pathol.

[42] Zarabian T, Noien S, Valizadeh S (2012). Talon cusp:case report and literature review. J Res Dent Sci.

[43] Kavitha S, Selvakumar H, Barathan R (2012). Mandibular talon cusp in primary lateral incisor: a rare case report. Case Rep Dent.

[44] Mohan RPS, Verma S, Singh U, Agarwal N, Ghanta S, Tyagi K (2013). Talon cusp in primary dentition:A case report. Int J Case Rep Imag.

[45] Bailey-Schmidt S (1995). The tri-form variant:I. Definition, classification and population distribution. Dent Anthrop Newsletter.

[46] Shey Z, Eytel R (1983). Clinical management of an unusual case of dens evaginatus in a maxillary central incisor. J Am Dent Assoc.

[47] Pledger DM, Roberts GJ (1989). Talon cusp:report of a case. Brit Dent J.

[48] Segura-Egea JJ, Jimenez-Rubio A, Velasco-Ortega E, Rios-Santos JV (2003). 2003a Talon cusp causing occlusal trauma and acute apical periodontitis:report of a case. Dent Traumatol.

